# Inhibition of O-GlcNAc Transferase Alters the Differentiation and Maturation Process of Human Monocyte Derived Dendritic Cells

**DOI:** 10.3390/cells10123312

**Published:** 2021-11-26

**Authors:** Matjaž Weiss, Marko Anderluh, Martina Gobec

**Affiliations:** 1 The Department of Pharmaceutical Chemistry, Faculty of Pharmacy, University of Ljubljana, 1000 Ljubljana, Slovenia; matjaz.weiss@ffa.uni-lj.si (M.W.); marko.anderluh@ffa.uni-lj.si (M.A.); 2 The Department of Clinical Biochemistry, Faculty of Pharmacy, University of Ljubljana, 1000 Ljubljana, Slovenia

**Keywords:** O-GlcNAcylation, O-GlcNAc transferase (OGT), monocyte derived DCs, OSMI-1, immunometabolism, OSMI-1

## Abstract

The O-GlcNAcylation is a posttranslational modification of proteins regulated by O-GlcNAc transferase (OGT) and O-GlcNAcase. These enzymes regulate the development, proliferation and function of cells, including the immune cells. Herein, we focused on the role of O-GlcNAcylation in human monocyte derived dendritic cells (moDCs). Our study suggests that inhibition of OGT modulates AKT and MEK/ERK pathways in moDCs. Changes were also observed in the expression levels of relevant surface markers, where reduced expression of CD80 and DC-SIGN, and increased expression of CD14, CD86 and HLA-DR occurred. We also noticed decreased IL-10 and increased IL-6 production, along with diminished endocytotic capacity of the cells, indicating that inhibition of O-GlcNAcylation hampers the transition of monocytes into immature DCs. Furthermore, the inhibition of OGT altered the maturation process of immature moDCs, since a CD14^med^DC-SIGN^low^HLA-DR^med^CD80^low^CD86^high^ profile was noticed when OGT inhibitor, OSMI-1, was present. To evaluate DCs ability to influence T cell differentiation and polarization, we co-cultured these cells. Surprisingly, the observed phenotypic changes of mature moDCs generated in the presence of OSMI-1 led to an increased proliferation of allogeneic T cells, while their polarization was not affected. Taken together, we confirm that shifting the O-GlcNAcylation status due to OGT inhibition alters the differentiation and function of moDCs in in vitro conditions.

## 1. Introduction

Dendritic cells (DCs) are a heterogeneous group of immune cells acting as messengers between the innate and adaptive immune response [1,2,3]. DCs are able to capture, process and present antigens [4,5,6,7] and in addition to antigen presentation, DCs mediate the appropriate effector or regulatory T cell immune responses by secreting several cytokines, growth factors, and delivering co-stimulatory signals [8,9]. Their unique ability to initiate, coordinate and regulate naïve T cell differentiation into distinct subsets including Th1, Th2 and Th17, as well as regulatory T cells, makes them the most efficient antigen presenting cells (APC) [4,8,9]. Thus, DCs play a key role in inducing and maintaining the T cell immune response and tolerance. DCs mature and migrate to secondary lymphoid organs upon activation signal via pattern recognition receptors (PRRs) expressed on DCs following PAMP (Pathogen-associated molecular pattern) and/or DAMP (Damage-associated molecular pattern) recognition [10,11,12,13,14]. Occupation of PRRs, i.e., Toll-like receptors (TLRs), nucleotide-binding oligomerization domain (NOD)-like receptors (NLRs), AIM2-like receptors (ALRs), retinoic acid-inducible gene I (RIG-I)-like receptors (RLRs) and C-type lectins, activates signalling pathways that change gene expression and metabolic demands [15]. It has been shown that these receptors are mainly signalling through the mitogen-activated protein kinase (MAPK), the NF-kB and IRF-3/7 pathways [13,15,16,17]. Additionally, the serine/threonine kinase mammalian target of rapamycin (mTOR) serves as a nutrient sensor. It regulates the process of glycolysis, which provides energy for highly demanding processes (e.g., differentiation of DCs) [6,18]. The metabolic effect of DC activation causes a shift from oxidative phosphorylation (OXPHOS) toward glycolytic processes and higher production of the tricarboxylic acid (TCA) cycle intermediates [19,20,21,22]. In addition, the metabolites of mTOR pathways play a significant role in epigenetic and transcriptional imprinting and consequently also define DC functions [18,22,23]. However, the discovery of cross-talk between PRRs suggests that their role in the immune system is more complex and sophisticated [13]. Manipulation of DC signalling pathways could be used to modify immune responses for therapeutic purposes [5,7,24,25].

The signalling pathways mentioned above include many tyrosine or serine kinases, which can be also O-GlcNAcylated [26,27,28,29,30]. The PI3K-Akt-mTOR signalling axis is an example of a signalling pathway known to be susceptible to O-GlcNAcylation and the cross-talk between PI3K/AKT/mTOR signalling and O-GlcNAcylation has been observed in several cell types [30,31]. O-GlcNAcylation is one of the posttranslational modifications (PTM) and is an important metabolic process in cells that needs to be carefully regulated [32] and its dysregulation is linked to several pathologies, including cancer, inappropriate immune responses and impairment of the nervous system [33,34,35,36,37]. O-β-*N*-acetylglucosaminyl transferase (OGT) is an enzyme that catalyses the transfer of *N*-acetylglucosamine from uridine diphosphate to serine and threonine residues of nuclear and cytoplasmic proteins, while O-GlcNAcase (OGA) removes O-GlcNAc moieties from proteins [33,38]. O-GlcNAcylation is therefore a dynamic process in the physiological environment that is regulated with both enzymes, the availability of UDP-GlcNAc nutrients [39], and posttranslational modification of target proteins [40] or enzymes themselves [40,41]. Previous work has shown that impaired protein O-GlcNAcylation affects protein functions [42], cell signalling [43,44], cell cycle [35], transcription [43] and also epigenetic factors [30]. The role of O-GlcNAcylation in immune cells has been studied in vitro in recent years. Impaired O-GlcNAcylation in macrophages [45,46,47], neutrophils [45], T cells [46,48,49] and B lymphocytes [50] lead to their inappropriate immune functions showing that O-GlcNAcylation plays a significant role in the immune system. Most immune cells need adequate O-GlcNAcylation for proper development and function [45,51]. Namely, reduced glycosylation of NFAT, NF-κB and c-Myc in T and B lymphocytes or NF-κB in macrophages resulted in loss of their function [45,46,51]. However, the role of O-GlcNAcylation in human dendritic cells has not been addressed yet. In this study, we examined the effects of the OGT inhibitor (OSMI-1) [40] in the human moDC differentiation and maturation process into immature and mature moDCs, respectively.

## 2. Results

### 2.1. OGT Inhibitor OSMI-1 Affects the MEK/ERK and mTOR/AKT Signalling Axis in Immature moDCs

**Figure 1:**
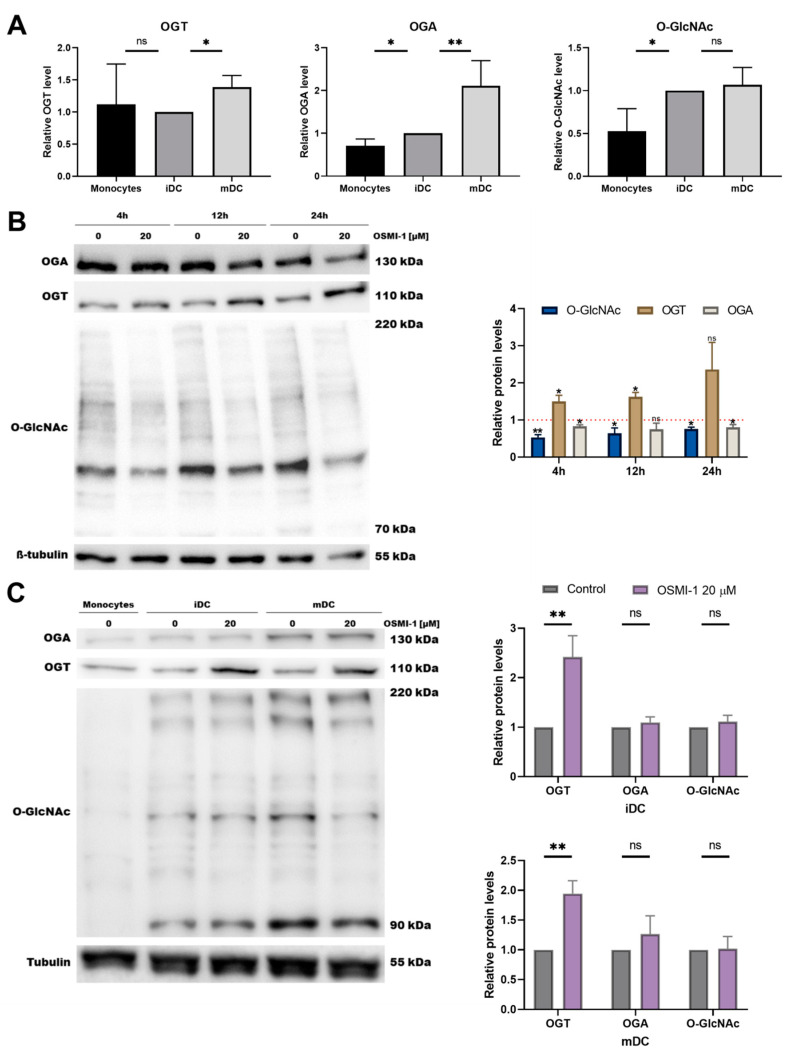
Protein levels of OGT, OGA and O-GlcNAcylated proteins. (**A**) Monocytes, immature and mature moDCs have distinct levels of OGT, OGA and O-GlcNAcylated proteins. Results are normalized to immature moDCs values and presented as relative protein expression ± SD (3–5 different donors). (**B**) Representative immunoblots for O-GlcNAcylated proteins, OGT and OGA in the presence or absence of 20 µM OSMI-1. Samples were collected after 4, 12 or 24 h from the start of the differentiation process. ß-tubulin was used as the loading control. Results are presented as relative protein levels to untreated cells ± SD (3 different donors). (**C**) Representative immunoblots for O-GlcNAcylated proteins, OGT and OGA in monocytes and immature or mature moDCs prepared in the presence or absence of 20 µM OSMI-1. Tubulin was used as the loading control. Results are presented as relative protein levels to untreated cells ± SD (4–5 different donors). Statistical significance between individual pairs was calculated using the ANOVA; *p*-value < 0.05 was considered statistically significant (* *p* < 0.05; ** *p* < 0.01; ns, nonsignificant). iDCs, immature moDCs; mDCs, mature moDCs.

The development and function of most immune cells depends on the appropriate profile of O-GlcNAcylated proteins. Since the main regulators of O-GlcNAcylation are OGT and OGA, we first examined their protein expression level in target cells (Figure 1A). The OGT level was similar in all three tested cell types, i.e., monocytes, immature moDCs, mature moDCs, while the expression of OGA was slightly increased in immature moDCs and was twice as high in mature moDCs compared to monocytes. The later somehow contradicts the observed level of O-GlcNAcylated proteins, which was higher in moDCs than in monocytes. Since OGA is responsible for O-GlcNAc cleavage, one would expect the lower degree of O-GlcNAcylation in case of higher OGA expression. On the other hand, higher level of O-GlcNAcylated proteins may cause a rebound in higher expression of OGA. In the following experiments we studied the impacts of an OGT inhibitor, OSMI-1, on moDC characteristics. Firstly, the moDC viability was evaluated after exposure to OSMI-1 for 6 or 8 days during the generation of iDCs and mDCs, respectively. In general, 20 µM OSMI-1 had no effect on cell viability (Figure S1). To clarify how OSMI-1 affects the relations between OGA/OGT expression and the status of O-GlcNAcylation, we examined their levels in the beginning of the differentiation process. As expected, after 4 h OSMI-1 caused a decrease in O-GlcNAcylated proteins. At a later time point, the effect faded due to compensatory mechanisms, which upregulated OGT and downregulated OGA levels (Figure 1B).

To explore the potential impact of in vitro OGT inhibition on the development and activation of moDCs, we next differentiated and matured monocytes in the presence or absence of 20 µM OSMI-1. In immature moDCs, where OSMI-1 was added during the 6 days of differentiation (immature OSMI-1-moDCs), a significant increase in OGT expression was observed, while the levels of either OGA or O-GlcNAcylated proteins remained the same (Figure 1C). A similar trend was also observed in mature moDCs, if immature OSMI-1-moDCs were further treated with OSMI-1 during the activation with rhGM-CSF, PGE_2_, rhIL-1ß, rhTNF-α and rhIL-6 (mature OSMI-1-moDCs).

**Figure 2:**
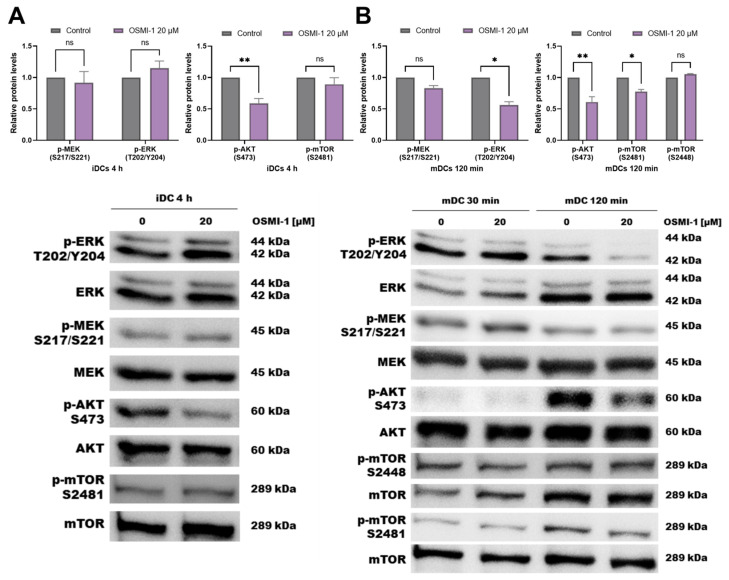
Characterisation of mTOR/AKT and MEK/ERK signalling pathways in the presence of OSMI-1. Representative immunoblots show the expression of phosphorylated and total ERK, MEK, AKT and mTOR in untreated or OSMI-1 treated immature (**A**) and mature moDCs (**B**) (3 different donors). The results in charts are presented as a ratio of phosphorylated/total protein levels normalized to untreated moDCs ± SD. Statistical significance between individual pairs was calculated using ANOVA; *p*-value < 0.05 was considered statistically significant (* *p* < 0.05; ** *p* < 0.01; ns, nonsignificant). iDCs, immature moDCs; mDCs, mature moDCs.

Since presence of OSMI-1 caused an increase in OGT expression in immature and mature OSMI-1-moDCs via a rebound loop [52,53], we next investigated the potential impact of these protein phenotypes on the signalling pathways associated with OGT involvement. These include the MEK/ERK and PI3K/AKT/mTOR signalling axis. We measured the intracellular levels of phosphorylated ERK, AKT, MEK and mTOR proteins in the presence or absence of OSMI-1 during the differentiation of immature moDCs and their activation towards mature moDCs. As depicted in Figure 2A, in the early phases of the differentiation process OSMI-1 significantly decreased the phosphorylation of AKT and mTOR (S2481), while no changes in the activation of ERK or MEK could be observed. Surprisingly, after 6 days of differentiation a shift in the phosphorylation status occurred, with a significant increase of phosphorylated ERK, MEK and AKT (Figure S2). On the other hand, if the OGT inhibitor was also present during the maturation process of immature OSMI-1-moDCs, decreased levels of phosphorylated ERK, MEK, AKT (S473) and mTOR (S2481) were detected (Figure 2B). The presence of OGT had no effect on the phosphorylation level at S2448 of mTOR, which remained intact in immature as well as in mature moDCs.

### 2.2. OSMI-1 Impairs the Differentiation and Activation/Maturation Process of moDCs

**Figure 3:**
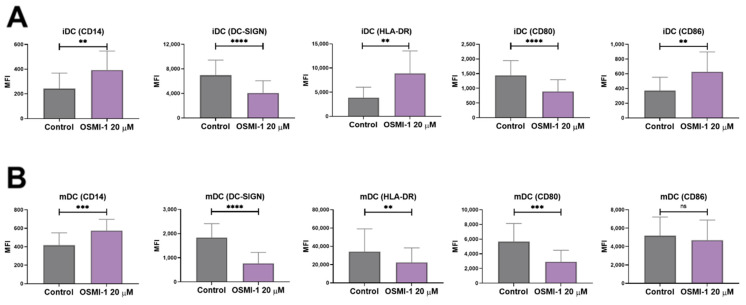
Characterisation of specific DC surface markers in the presence of OSMI-1. (**A**) Immature moDCs differentiated from monocytes for 6 days in the presence or absence of OSMI-1. (**B**) Mature moDCs were generated by the addition of the cytokine cocktail for 2 days either from immature moDCs (control) or immature OSMI-1-moDC. The activation of later was carried out in the presence of OSMI-1. Shown are mean MFI values ± SD of *n* = 8–11 independent experiments. Statistical significance between individual pairs was calculated using Student’s *t*-test; *p*-value < 0.05 was considered statistically significant (** *p* < 0.01; *** *p* < 0.001; **** *p* < 0.001; ns, nonsignificant). iDCs, immature moDCs; mDCs, mature moDCs.

The communication between DCs and other (immune) cells is mediated by surface proteins, which also serve as markers for their identification. Thus, any change in their expression can have physiological or pathological consequences. To identify and characterize the potential impact of OSMI-1 on the expression of surface markers specific for DCs, we investigated the levels of CD14, CD80, CD86, HLA-DR and DC-SIGN in immature and mature moDCs. As depicted in Figure 3, OSMI-1 caused a pronounced shift in the expression of several surface proteins compared to control cells. Namely, if immature moDCs were generated in the presence of OSMI-1, down-regulation of the integrin receptor DC-SIGN and the co-stimulatory molecule CD80 was observed, while the expression of CD14, CD86 and HLA-DR significantly increased (Figure 3A). In addition, OSMI-1 also altered the maturation/activation of immature OSMI-1-moDCs stimulated with the cytokine cocktail (rhGM-CSF, PGE_2_, rhIL-1ß, rhTNF-α, rhIL-6) (Figure 3B), where DC-SIGN, HLA-DR and CD80 were downregulated, while CD14 was upregulated.

### 2.3. OGT Inhibition Leads to Hampered Endocytosis and Affects the Release of IL-6 and IL-10 in Immature moDCs

**Figure 4:**
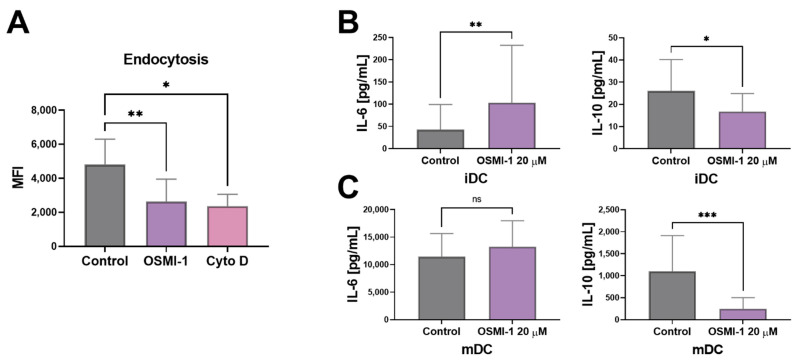
The impact of OSMI-1 on endocytosis and the release of cytokines. (**A**) Control or immature OSMI-1-moDCs were incubated with FITC-labelled dextran particles (molecular weight ≈ 40.000 Da) for 1 h. Cells incubated with cytochalasin D were used for a negative control. Endocytosis of FITC-conjugated dextran particles was determined by flow cytometry and the subtraction of MFIs of FITC positive cells at 37 °C with the MFI values obtained at 4 °C. Bars present the mean MFI values ± SD of six independent experiments. Cytokines concentration measured by CBA method in supernatants of untreated or OSMI-1 treated immature (**B**) and mature (**C**) moDCs, on day 6 or 8, respectively. Shown are mean concentrations ± SD of four to seven independent experiments. Statistical significance between individual pairs was calculated using Student’s *t*-test; *p*-value < 0.05 was considered statistically significant (* *p* < 0.05; ** *p* < 0.01; *** *p* < 0.001; ns, nonsignificant). iDCs, immature moDCs; mDCs, mature moDCs; Cyto D, cytochalasin D.

DCs are the professional antigen presenting cells that capture antigens through different mechanisms, including the fluid phase (FPE) or clathrin-mediated endocytosis (CME). The latter depends on the expression of specific carbohydrate receptors, including DC-SIGN, langerin or the mannose receptor (CD206) [54,55]. Since the expression of DC-SIGN was significantly diminished if OGT was inhibited, we next addressed the endocytotic capacity of DCs. We observed that the presence of OSMI-1 during the monocyte differentiation process led to decreased endocytotic capacity of immature OSMI-1-moDCs compared to non-treated control cells (Figure 4A). In case of mature moDCs, the uptake of dextran particles conjugates was already low in non-treated DCs, as expected, and the presence of OSMI-1 had no additional effects (data not shown).

Since soluble factors of the immune system play an important role in mediating cell-cell communication, we next evaluated whether OSMI-1 alters the cytokine secretion profile of IL-6, IL-10, IL-12p70, IL-8, IL-1ß or TNF-α in moDCs. The inhibitor caused a decreased production of IL-10 and an increase in IL-6 levels in immature moDCs (Figure 4B). A similar trend was observed in mature moDCs, however the changes in IL-6 were not significant (Figure 4C). The production of IL-1ß, IL-8, TNF-α and IL-12p70 was not affected (data not shown).

### 2.4. OSMI-1 Treated Mature moDCs Exert Increased Capacity of Promoting Allogeneic T Cell Proliferation

**Figure 5:**
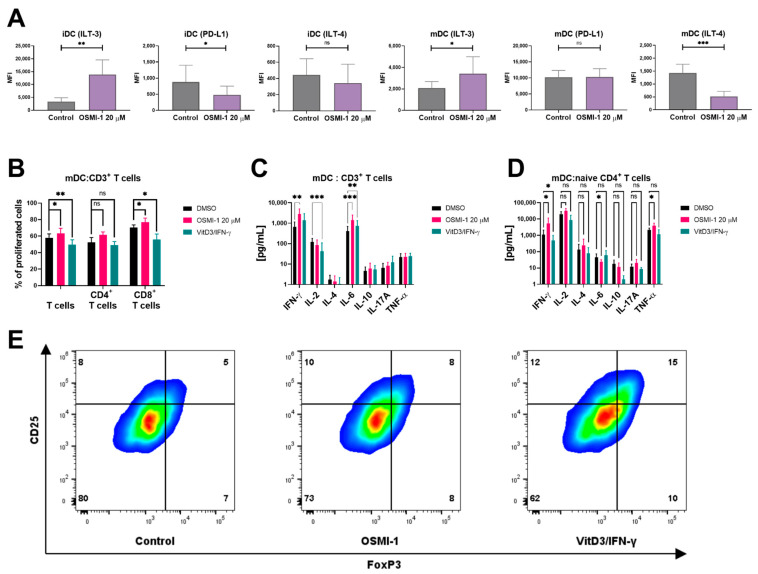
Characterisation of OSMI-1 treated mature moDCs impacts on allogeneic T lymphocytes. (**A**) Characterisation of moDC surface markers after exposure to OSMI-1. Shown are mean MFI values ± SD from 8–11 independent experiments. (**B**) Representative T-cell proliferation after 6 days of co-culture of T cells (CD3^+^) and mature moDCs. T cells were cultured with either untreated or OSMI-1 or VitD_3_/IFN-γ treated mature moDCs. (**C**) Cytokine concentrations in supernatants taken from co-cultures of CD3^+^cells:moDCs after 6 days. Supernatants were analysed for the presence of IL-2, IL-4, IL-6, IL-10, IL-17A, IFN-γ and TNF-α. The results are expressed as means ± SD of four independent experiments. (**D**) The T cell-polarizing capacity of mature moDCs was determined by co-culturing differently treated mature moDCs with naïve allogeneic CD4^+^CD45RA^+^ T cells. After 14 days, the supernatants were sampled and analysed for the presence of IL-2, IL-4, IL-6, IL-10, IL-17A, IFN-γ and TNF-α. The results are expressed as means ± SD of two to three independent experiments. (**E**) The T cell-polarizing capacity of mature moDCs toward regulatory T cells (Tregs) was determined by co-culturing differently treated mature moDCs with naïve allogeneic CD4^+^CD45RA^+^ T cells. After 14 days, the cells were washed and analysed by flow cytometry for the expression of CD25 and FoxP3. Representative results of CD25 and FoxP3 expression are presented as dot plots. Statistical significance between individual pairs was calculated using Student’s *t*-test; *p*-value < 0.05 was considered statistically significant (* *p* < 0.05; ** *p* < 0.01; *** *p* < 0.001; ns, nonsignificant). iDCs, immature moDCs; mDCs, mature moDCs.

The main role of DCs in the immune system is the formation of the immune synapse with T cells to induce their antigen-specific activation. Therefore, we next addressed the capability of OSMI-1 treated mature moDCs to induce allogeneic T cell proliferation. Based on previous results, we expected a more tolerogenic profile of such moDCs, therefore we firstly investigated the expression of inhibitory molecules CD274 (PD-L1), CD85k (ILT-3) and CD85d (ILT-4) on the surface of both OSMI-1 treated immature and mature moDCs. As depicted in Figure 5A, DCs exposed to OSMI-1 exhibit pronounced up-regulation of inhibitory receptor immunoglobulin like transcript 3 (ILT-3) molecules, which can independently inhibit T cell activation. Surprisingly, OSMI-1 caused down-regulation of two other inhibitory molecules, the program death-ligand 1 (PD-L1) and immunoglobulin like transcript 4 (ILT4) on both immature and mature moDCs. As a positive control for a DC tolerogenic phenotype, immature moDCs exposed to VitD_3_/IFN-γ were used. In accordance with previous reports, an up-regulation of ILT-3 and ILT-4 expression was detected on these controls (Figure S3). To determine how these phenotypic changes translate into the functionality of DCs, we next used mature moDCs as stimulators of allogeneic T lymphocytes (Figure 5B,C). The proliferation rate of T cells was determined after 6 days of co-culture. An increase in the proliferation rate of T cells was observed if co-cultures were prepared with mature moDCs, which were differentiated and activated in the presence of OSMI-1. Again, co-cultures with VitD_3_/IFN-γ treated mature moDCs, were used as control presenting a tolerogenic DC phenotype (tolDCs). As expected, the presence of tolDCs supressed the proliferation of T cells. Further analysis of T cell proliferation revealed a statistically significant increase in the number of CD8^+^ T cells upon co-culture with OSMI-1 treated moDCs as compared with control cells. A similar trend was also observed with CD4^+^ cells; however, it was not statistically significant. Co-cultures produced high levels of IFN-γ and IL-6, and moderate levels of IL-2. The concentrations of IFN-γ and IL-6 were increased, while the IL-2 release was down-regulated in co-cultures containing OSMI-1 treated mature moDCs in comparison to control cells (Figure 5C). Additionally, we explored the polarization of naïve CD4^+^ T cells with the analysis Th1/Th2/Th17 cytokines (Figure 5D). The co-cultures of naïve CD4^+^ T cells were prepared with either control, OSMI-1 or VitD_3_/IFN-γ treated mature moDCs. Supernatants were collected after 14 days and additional 5 h stimulation with PMA/ionomycin.

In co-cultures of mature OSMI-1-moDCs and CD4^+^ cells an increase in secretion of IFN-γ and TNF-α was observed, while IL-6 decreased. No significant changes were determined for IL-2, IL-4, IL-10 or IL-17A if compared to control. In the media of co-cultures of VitD_3_/IFN-γ treated mature moDCs and CD4^+^ cells a contrary trend was noticed, however, only the data for IFN-γ was statistically significant. The obtained cytokine profile of co-cultures with mature OSMI-1-moDCs indicates polarization of Th0 to Th1, which typically secret sizable amounts of IFN-γ, TNF-α and IL-2. The opposite trend in cytokine release observed in co-cultures of naïve T cells with VitD_3_/IFN-γ treated mature moDCs, indicates polarization of T cells into regulatory cells (Tregs). Indeed, these T cells had a shift of the phenotype markers from CD25^low^FoxP3^low^ to CD25^high^FoxP3^high^, which is characteristic for Tregs. However, this phenotype was not observed in T cells that were co-cultured with mature OSMI-1-moDCs, confirming that OGT inhibition in DCs does not induce polarization into Tregs (Figure 5E).

## 3. Discussion

O-GlcNAcynation is a metabolically dependant process integrated in many signalling pathways via the crosstalk with kinases [26,27,56,57]. It has been shown that imbalances in OGT activity leads to cell dysfunction. This also includes the immune cells, where disturbed O-GlcNAcylation in macrophages [45,46,47], neutrophils [45], T cells [46,48,49] and B lymphocytes [50], led to their altered functionality. The paradox of O-GlcNAcylation is that under acute stress conditions, O-GlcNAc accumulation is protective and allows cells to survive. However, under chronic conditions increased O-GlcNAcylation disrupts crucial signalling pathways, transcriptional processes and leads to cell damage [58,59]. Moreover, it has been shown that modulators of O-GlcNAcylation have different effects on different immune cells. The type of insult is probably the main factor that determines whether their role will be pro- or anti-inflammatory [60]. However, the role of OGT in dendritic cells, which are the most important link between the innate and adaptive immunity, has not been addressed to date. Herein, we demonstrate that OGT inhibition hampers the transition of monocytes into immature moDCs and directs their subsequent maturation process into an alternatively maturated phenotype.

We investigated the function of OGT with the use of its inhibitor, OSMI-1. Firstly, the effects of OSMI-1 on mTOR-AKT signalling was addressed, since the results of previous research suggested that increased O-GlcNAcylation together with the up-regulated OGT enhances AKT activation [30,61]. The differentiation process of monocytes into immature moDCs is known to involve the PI3K-mTOR-AKT signalling axis, which regulates development, survival and function of human DCs [62]. Herein, we demonstrate that the presence of OSMI-1 during the differentiation process decreased the phosphorylation at the S2481 site of the mTOR, indicating that the functionality of mTORC2 was affected. It is known that fully activated AKT requires phosphorylation at T308 and at S473 by PDK-1 and mTORC2, respectively [63]. If phosphorylation at S473 is lacking, the AKT activity is greatly diminished [64]. Indeed, we also found that reduced mTORC2 activation reflected in diminished AKT phosphorylation. This phenotype was present at the beginning of the differentiation process along with decreased O-GlcNAcylation status. At the end of differentiation, the O-GlcNAcylation status returned to normal levels, due to compensation, and AKT became hyperphosphorylated. The PI3K-AKT hyperactivation in DCs is associated with increased expression of MHC class II and co-stimulatory molecules, as well as enhanced production of cytokines (including IL-6) [65,66]. We also observed such phenotype in immature OSMI-1-moDCs. It has been reported that modulation of O-GlcNAcylation can either decrease or increase phosphorylation of AKT [30,67]. Thus, it is not surprising that inhibition of OGT had an opposite effect on the AKT phosphorylation status in monocytes and moDCs, especially if we take into consideration that these two cell types differ in phenotype and functions. Of note, the dual role of O-GlcNAcylation in the context of pro- or anti-inflammatory effect is well known and is nicely presented in macrophages and T cells. Hyper O-GlcNAcylation is predicted to boost the pro-inflammatory function of M1 macrophages, while it enhances the anti-inflammatory response of M2 [68]. In T cells, it has been shown that adequate O-GlcNAcylation is crucial for lineage stability and effector function in Treg cells, while it enhanced the pro-inflammatory role of Th1. [49,69].

Among the signalling pathways that are crucial in shaping functional DCs is also the MEK/ERK axis, which tightly regulates secretion of pro-inflammatory cytokines [70]. It has been recently demonstrated that the reduced O-GlcNAcylation or OGT knockdown supresses ERK1/2 activation in neutrophils and gastric cancer cells [71,72]. Herein, we show that OGT inhibition affects the MAPK cascade differently in immature as in mature moDCs. Namely, the presence of OSMI-1 during the differentiation process of monocytes to immature DCs led to activation of the MAPK pathway (as witnessed by increased levels of phosphorylated ERK1/2 and MEK at day 6). On the other hand, if the inhibitor was also present during the maturation of immature moDCs to mature moDCs, reduction of the phosphorylation status was observed compared to untreated cells. This indicates a dual role of OGT, which depends on the developmental state of DCs. Along with the observed increase of the pro-inflammatory cytokine IL-6 and reduced release of anti-inflammatory IL-10, we can conclude that OSMI-1 induces a pro-inflammatory phenotype in immature moDCs. Of note, similar results were obtained in macrophages where OGT depletion increased TNF-α and IL-6 secretion and promoted inflammation [73]. Investigation of relevant surface markers also revealed an altered expression profile when DCs were generated in the presence of OSMI-1. The latter prevented the downregulation of CD14 and caused an up-regulation of CD86, HLA-DR and ILT-3, while the expression of DC-SIGN and ILT-4 remained significantly low. The regulation of these markers is complex and often ambiguous, but such a phenotypic profile, along with the altered secretion of IL-6 and IL-10, indicates differentiation of monocytes into an alternative population of immature moDCs with impaired endocytic capacity. Since the OGT inhibition led to such a distinct phenotype of immature moDCs, we next investigated if this observation also translates to mature moDCs. As expected, when OSMI-1 was present during the maturation process, a shift in surface markers was observed, implying an alternatively matured population of mature moDCs, being CD14^med^DC-SIGN^low^HLA-DR^med^CD80^low^CD86^high^ILT-3^high^ILT-4^low^ with a decreased capacity to secrete IL-10. Activation and polarization of T cells depends on the expression of co-stimulatory and inhibitory molecules on DCs, their capacity of antigen presentation by HLA-DR and presence of cytokines in the microenvironment. These parameters were altered in OSMI-1 treated moDCs, thus we next evaluated if the alternatively matured moDCs were able to elicit allogeneic stimulation of T cells. Firstly, mature moDCs were used in co-cultures with allogeneic lymphocytes. An increase in proliferation rate of T cells was observed if co-cultures were prepared with OSMI-1 pre-treated mature moDCs. Flow cytometry analysis of proliferating T cells showed a statistically significant increase in the number of CD8^+^ T cells. A similar trend was also observed with CD4^+^ cells; however, it was not statistically significant. In addition, these co-cultures produced higher levels of IFN-γ and IL-6 compared to co-cultures where untreated mature moDCs were used. These observations were unexpected, since we predicted that the decreased expression of HLA-DR and co-stimulatory molecules in mature moDCs generated in the presence of OSMI-1 would hamper their ability to induce T cell proliferation. Apparently, the expression of these molecules was still high enough. Moreover, decreased IL-10 secretion along with lower expression of inhibitory receptor ILT-4, seem to have helped to accelerate T cell proliferation. To delineate the polarization of T cells, we prepared co-cultures of mature moDCs with naïve CD4^+^ T cells. Normally, IL-12 secreted from mature moDCs induces polarization of Th0 into Th1 cells, which are characterised as IFN-γ, TNF-α and IL-2 secreting cells. In our case, moDCs did not secrete IL-12, probably due to the cytokine cocktail used for activation. The latter namely contains PGE_2_, which is known to inhibit production of several chemokines and cytokines, including IL-12 [74]. However, the analysis of supernatants from allogeneic co-cultures of T cells and mature OSMI-1-moDCs revealed high levels of IFN-γ, TNF-α and IL-2 suggesting polarization into Th1 cells. A similar cytokine profile was also found in co-cultures of T cells with VitD_3_/IFN-γ pre-treated mature moDCs (tolDCs). Mature moDCs generated in the presence of VitD_3_/IFN-γ are known to induce polarization of naïve T cells into regulatory T cells (Tregs) [75]. Based on the similarity of results obtained from our co-cultures, we assumed that the mature moDCs generated in the presence of OSMI-1 would cause polarization of naïve T cells into Tregs. For the latter a shift in the expression from CD25^low^FoxP3^low^ to CD25^high^FoxP3^high^ is characteristic. Surprisingly, this phenotype was not observed on T cells from co-cultures with mature OSMI-1-moDCs, but was present in positive controls where tolDCs were used. However, one must point out, that the Treg profile and cytokine profile obtained from co-cultures of T cells with tolDCs was not extensively pronounced. This could be attributed to the cytokine cocktail used for the activation, which consisted of rhGM-CSF, PGE_2_, rhIL-1ß, rhTNF-α and rhIL-6. Usually, in experiments where tolDCs are generated in the presence of VitD_3_/IFN-γ alone or the activation is carried out with LPS [75,76].

To conclude, reports show that a shifted O-GlcNAcylation status is present in several pathologies [34,36,51]. For example, in mice with induced inflammatory bowel disease increased O-GlcNAcylation was noticed. Such phenotype was also observed in intestinal epithelial tissues of patients with Crohn’s disease [77]. In malignancies it is well known that enhanced levels of O-GlcNAcylation are present. Primary pre-B acute lymphocytic leukemia (pre-B-ALL) cells have high levels of O-GlcNAcylated proteins and upregulated OGT accompanied with an overactivated PI3K/AKT/c-Myc axis. Moreover, in cell lines the suppression of OGT reduced the cell proliferation rate and induced apoptosis [61]. It is becoming clear that dysregulation of O-GlcNAcylation can either play a pro- or anti-inflammatory role, which depends on the type or developmental stage of the immune cell in question [60,78]. In the case of moDCs, we show that OGT inhibition affects several signalling pathways during the differentiation and maturation of moDCs. The end result is an alternative type of moDCs with altered functionalities in terms of endocytosis, T cell proliferation stimulating capacity and T cell polarization. These primary observations pave the way for future studies, where the role of OGT can be further evaluated as a potential target for therapeutic interventions in diseases linked to disrupted O-GlcNAcylation status. However, the role of OGT should be investigated with care and in respect to the immune cell type, due to its involvement in several intracellular processes.

## 4. Materials and Methods

### 4.1. Isolation of Monocytes, T Cells and Naïve CD4^+^ T Cells from PBMCs

Buffy coats from venous blood of anonymized healthy donors were purchased from the Blood Transfusion Centre of Slovenia. Peripheral blood mononuclear cells (PBMCs) were isolated using Lymphoprep (Stemcell Technologies, Vancouver, BC, Canada). The cells were washed twice with phosphate-buffered saline (PBS), counted, and used as a source for the immunomagnetic negative isolation of CD14^+^ cells (EasySep, Stemcell Technologies) or CD4^+^ naïve T cells (MojoSort, Biolegend, San Diego, CA, USA). The purity of CD14^+^ and naïve T cells was greater than 85 and 96%, respectively, as determined by flow cytometry. CD3^+^ T cells were obtained by negative selection using RosetteSep Human T cell enrichment cocktail (Stemcell Technologies) and by density centrifugation using Lymphoprep. The cells were washed twice with PBS. The purity of T cells was greater than 95%, as determined by flow cytometry.

### 4.2. Generation of Monocyte-Derived Dendritic Cells (moDCs)

Monocytes were cultured in RPMI 1640 (Sigma-Aldrich, St. Louis, MO, USA) medium, supplemented with 10% fetal bovine serum (Gibco, Life Technologies, Paisley, UK), 50 U/mL penicillin and 50 µg/mL streptomycin (Sigma-Aldrich), 800 U/mL of rhGM-CSF and 1000 U/mL of rhIL-4 (both Biolegend) at 0.5–1 × 10^6^ cells/mL. Cells were cultured in a humidified incubator at 37 °C and 5% CO_2_. On day 3, half of the medium was exchanged with the addition of double quantities of rhGMCSF and rhIL-4. In some instances, 20 μM OSMI-1 was added to monocytes at the start of differentiation. After 6 days, non-adherent, immature moDCs were harvested and characterized by flow cytometry. For maturation, immature moDCs were counted and re-suspended in RPMI-1640 medium containing 1000 IU/mL of rhGM-CSF, 1 µg/mL of PGE_2_ (Cayman Chemical, Ann Arbor, MI, USA), 200 IU/mL of rhIL-1ß (Invivogen, San Diego, CA, USA), 1000 IU/mL of rhTNF-α (eBiosceience, ThermoFisher Scientific, Waltham, MA, USA), and 1000 IU/mL of rhIL-6 (Biolegend) and matured for 2 additional days [79]. In some instances, 10 ng/mL of Vitamin D_3_ (Sigma-Aldrich) together with 500 IU/mL rhIFN-γ (Biolegend) or 20 μM OSMI-1 (results depicted in Figure S4) were added to untreated immature moDCs or 20 μM OSMI-1 was added to immature OSMI-1-moDCs at the start of maturation. After 8 days, non-adherent, mature moDCs were harvested and characterized by flow cytometry.

### 4.3. Immunophenotyping of moDCs

The expression of surface markers was characterized by flow cytometry and the use of specific antibodies. On day 0, 6 or 8, non-adherent cells were harvested and collected by centrifugation. The cells were re-suspended in a solution of selected antibodies (PBS, 2mM EDTA, 0.5% BSA) and incubated at 4 °C for 20 min in the dark. Afterwards, 400 µL of PBS was added and the samples were centrifuged at 300× *g* at 4 °C for 5 min. The cells were then re-suspended in 400 µL of PBS and analysed on Attune NxT flow cytometer (Life Technologies, Carlsbad, CA, USA). The following monoclonal antibodies were used: anti-CD14 Pacific Orange (ThermoFisher Scientific), anti-DC-SIGN APC/Fire 750, anti-CD80 AlexaFluor 488, anti-CD86 Pacific Blue, anti-HLA-DR PerCP, anti-CD85k APC, anti-CD274 PE (All from Biolegend), anti-CD85k FITC, anti-CD274 APC, anti-CD85d PE (all from Miltenyi Biotec, North Rhine-Westphalia, Germany). For isotype control IgG1, IgG2a and IgG2b cocktail was used (all from Biolegend). The results are expressed as median fluorescence intensity (MFI) values.

### 4.4. Allogeneic T Cell Proliferation Assay

The proliferation of T cells was determined using the CellTrace^TM^ Cell Proliferation Kit (Invitrogen Molecular Probes, Eugene, OR, USA) in accordance with the manufacturer’s instructions. T cells were re-suspended in PBS at 2 × 10^6^ cells per mL and incubated with 5 μM CFSE dye for 10 min at 37 °C. Cells were then washed two times with culture medium, re-suspended and plated on U-bottom 96-well plate at 15 × 10^4^ cells per well. Allogeneic mature moDCs were washed with PBS, counted, and 1.5 × 10^4^ cells per well were added to CFSE-labelled T cells and incubated for 6 days (the ratio T cell: moDC was 10:1). All assays had a negative control (T cells alone) and positive control (addition of 10 μg/mL of PHA (Sigma-Aldrich)). After 6 days of incubation supernatants were collected and analysed for the presence of IL-2, IL-4, IL-6, IL-10, IL-17A, IFN-γ and TNF-α. The remaining T cells were analysed by flow cytometry to determine their proliferation rate.

### 4.5. T Cell Polarization

Isolated naïve CD4^+^ T cells were plated in U-bottom 96 well plate at 15 × 10^4^ cells per well. Mature moDCs were washed with PBS, counted, and added to naïve T cells at 1.5 × 10^4^ cells per well and incubated for 6 days. The remaining cells were washed and re-suspended in RPMI 1640 medium supplemented with 10% fetal bovine serum, 50 U/mL penicillin, 50 µg/mL streptomycin and 100 IU/mL rhIL-2, and plated in a 96 well plate. Every second day half of the medium was replaced with the fresh one. On day 10, the cells were transferred in 48 well plates. On day 14, the supernatants were collected and the cells were washed and counted. Cells (5 × 10^5^) were used for characterisation of Tregs. Remaining cells (5 × 10^5^) were stimulated with 1.4 µM ionomycin (Sigma-Aldrich) and 16.2 nM PMA (Sigma-Aldrich) for 5 h. Supernatants were collected for cytokine detection at the end of stimulation.

### 4.6. Immunophenotyping of T Cells

The expression of surface markers was characterized by flow cytometry and the use of specific antibodies. On day 6 (T cell proliferation) or 14 (T cell polarization), the cells were harvested and collected by centrifugation. To determine T cell proliferation rate the cells were re-suspended in a solution of selected antibodies (PBS, 2mM EDTA, 0.5% BSA) and incubated at 4 °C for 20 min in the dark. Afterwards, 400 µL of PBS was added and the samples were centrifuged at 300× *g* at 4 °C for 5 min. The cells were then re-suspended in 400 µL of PBS and analysed on Attune NxT flow cytometer. The following monoclonal antibodies were used: anti-CD4 APC; anti-CD8 PE-Cy5 (both BD Biosciences, San Jose, CA, USA). The results are expressed as percentage of divided cells. To determine percentage of Treg, the Treg Detection Kit was used according to manufacturer’s instructions (Miltenyi Biotec). In brief, the cells were re-suspended in a solution of anti-CD4 VioGreen, anti-CD25 VioBright 515, anti-CD127 PE (PBS, 2mM EDTA, 0.5% BSA) and incubated at 4 °C for 10 min in the dark. Afterwards, the cells were washed and fixed at 4 °C for 30 min. Fixed cells were washed and permeabilised together with tandem signal enhancer and anti-FoxP3 Vio667 at 4 °C for 30 min in the dark. At the end the cells were washed and re-suspended in 400 µL of PBS and analysed on flow cytometer. The results are expressed as percentage of CD25^+^FoxP3^+^ cells.

### 4.7. Endocytosis Assay

Endocytosis was assessed by flow cytometry using FITC-conjugated dextran particles (molecular weight ≈ 40.000 Da; Life Technologies). Immature moDCs were harvested and centrifuged at 300× *g* for 7 min at 4 °C. In brief, 1 × 10^5^ cells were incubated in RPMI-1640 with 1 mg/mL of FITC conjugated dextran particles for 1 h either at 37 °C or 4 °C. For negative control, the cells were incubated at room temperature without the FITC-dextran. Cells pre-treated with 5 µM cytochalasin D (Sigma-Aldrich) for 1h were used as a negative control. Afterwards, the samples were washed three times with 2% BSA in PBS and centrifuged at 300× *g* at 4 °C for 10 min. The cell pellets were resuspended in PBS and then analysed using the flow cytometer. Endocytosis of FITC-conjugated dextran particles was determined by the subtraction of MFIs of FITC positive cells at 37 °C with the MFI values obtained at 4 °C.

### 4.8. Immunoblotting

Monocytes, immature and mature moDCs were cultured at a density of 1 × 10^6^ cells per mL and treated with compounds of interest or corresponding vehicle. At the defined time periods (O-GlcNAcylation status after 4, 12 and 24 h of initiated differentiation; signalling pathways after 4 h after start of differentiation or 30 and 120 min after start of maturation; the protein levels in non-treated cells on day 0, 6 and 8), 2 × 10^6^ cells were harvested and centrifuged at 3000 rpm for 5 min. Afterwards, the cells were re-suspended in ice-cold PBS, and centrifuged at 3000 rpm for 5 min. Cell pellets were lysed on ice using modified RIPA buffer, consisting of 50 mM Tris–HCl, pH 8.0, 150 mM NaCl, 1% NP-40, 0.5% Na-deoxycholate, 0.1% SDS, 1 mM EDTA, 1× Halt Phosphatase inhibitor cocktail and 1× Halt Protease inhibitor cocktail (Thermo Scientific). Then, the lysates were sonicated, rocked on ice for 30 min, and centrifuged at 15,000× *g* at 4 °C for 20 min. The samples containing 20 μg of protein were denaturated at 96 °C for 5 min in a sample loading buffer (3% SDS, 10% glycerol, 62.5 mM Tris–HCl, pH 6.8, 5% 2-mercaptoethanol, 0.1% bromphenol blue) and loaded on SDS-polyacrylamide gels. Electrophoresis was carried out in Tris-glycin buffer at 100 V, followed by wet transfer to nitrocellulose membranes (GE Healthcare Life Science, Uppsala, Sweden) or dry transfer to PVDF membranes using the iBlot2 (Invitrogen, Waltham, MA, USA). The SeeBlue^®^ Plus2 pre-stained reagent (Invitrogen) was used to determine the molecular weights of separated proteins. Nonspecific binding sites were blocked for 1 h at room temperature in 3% bovine serum albumin (Sigma-Aldrich) in tTBS (TBS, 0.1% Tween; Sigma-Aldrich). The membranes were then washed and incubated overnight at 4 °C with gentle stirring in a solution containing appropriate primary antibodies. On the next day, the membranes were washed three times with 0.1% Tween in TBS and incubated for 1 h at room temperature with the corresponding dilution of a secondary antibody conjugated to horseradish peroxidase (Cell Signaling Technology, Danvers, MA, USA) in a 5% solution of skim milk powder (Merck, Kenilworth, NJ, USA) (TBS, 0.1% Tween). After incubation, the membranes were washed 5-times in 0.1% Tween in TBS and then the SuperSignal West Femto substrate (ThermoScientific) was added. The chemiluminescent signal was acquired on the Uvitec Cambridge Alliance chemiluminometer (Uvitec, Lodi, NJ, USA). The band intensities were quantified using the Uvitec Imager. To ensure the equal loading of proteins, the membranes were stripped with a stripping buffer (100 mM 2-mercaptoethanol, 2% SDS, and 62.5 mM Tris/HCl, pH = 6.8) for 45 min at 50 °C and re-probed with antibodies as described above.

The antibodies and their dilutions used were as follows: anti-O-GlcNAcylation (CTD110.6; 1:1000; BioLegend), anti-OGA (HPA036141; 1:1000; Sigma-Aldrich), anti-OGT (24083; 1:1000; Cell Signaling Technology), anti-p-ERK1/2 (9101; 1:1000; Cell Signaling Technology), anti-p-MEK1/2 (9154; 1:1000; Cell Signaling Technology), anti-p-AKT (9271; 1:500; Cell Signaling Technology), anti-p-mTOR (5536; 1:1000; Cell Signaling Technology), anti-p-mTOR (2974; 1:800; Cell Signaling Technology), anti-ERK1 (sc-93; 1:1000; SantaCruz, Dallas, TX, USA), anti-ERK2 (sc-154; 1:5000; SantaCruz), anti-MEK1/2 (8727; 1:1000; Cell Signaling Technology), anti-AKT (4685; 1:1000; Cell Signaling Technology), anti-mTOR (2983S; 1:1000; Cell Signaling Technology), anti-ß-actin (A5316; 1:7000; Sigma-Aldrich), anti-tubulin (2148; 1:1000; Cell Signaling Technology), anti-ß-tubulin (2146; 1:1000; Cell Signaling Technology), anti-mouse IgG-HRP (7076, 1:10000; Cell Signaling Technology) and anti-rabbit IgG-HRP (7074; 1:10000; Cell Signaling Technology).

### 4.9. Cytokine Detection

Cell-free supernatants were obtained by centrifugation at 1200 rpm for 5 min and stored at −80 °C until measurement. Cytokine concentrations were assessed by the Human Inflammatory or the Human Th1/Th2/Th17 Cytometric Bead Array (CBA) kits (BD Biosciences) according to the manufacturer’s protocol. The data were analysed with FlowJo software. The results are expressed in pg/mL.

### 4.10. Statistical Analysis

Statistical analyses were performed using GraphPad Prism 8.2.1 or 9.2.0, and the data were evaluated using paired Student’s *t*-tests or 2-way ANOVA; *p* < 0.05 was considered to be indicative of statistical significance (* *p* < 0.05; ** *p* < 0.01; *** *p* < 0.001; **** *p* < 0.001).

## Data Availability

Raw data available upon request to corresponding author.

## Supplementary Materials

The following are available online at https://www.mdpi.com/article/10.3390/cells10123312/s1, Figure S1: Viability of moDCs, Figure S2: Characterisation of mTOR/AKT and MEK/ERK signalling pathways in the presence of OSMI-1, Figure S3: Characterisation of specific DC surface markers in the presence of Vitamin D3 together with IFN-γ (tolDCs). Figure S4: Characterisation of specific DC surface markers in the presence of OSMI-1 during maturation.

## References

[1] Eisenbarth S.C. (2019). Dendritic cell subsets in T cell programming: Location dictates function. Nat. Rev. Immunol..

[2] Mildner A., Jung S. (2014). Development and function of dendritic cell subsets. Immunity.

[3] Clark G.J., Silveira P.A., Hogarth P.M., Hart D.N.J. (2019). The cell surface phenotype of human dendritic cells. Semin. Cell Dev. Biol..

[4] Banchereau J., Briere F., Caux C., Davoust J., Lebecque S., Liu Y.-J., Pulendran B., Palucka K. (2000). Immunobiology of Dendritic Cells. Annu. Rev. Immunol..

[5] Mackern-Oberti J.P., Llanos C., Vega F., Salazar-Onfray F., Riedel C.A., Bueno S.M., Kalergis A.M. (2015). Role of dendritic cells in the initiation, progress and modulation of systemic autoimmune diseases. Autoimmun. Rev..

[6] Patente T.A., Pelgrom L.R., Everts B. (2019). Dendritic cells are what they eat: How their metabolism shapes T helper cell polarization. Curr. Opin. Immunol..

[7] Waisman A., Lukas D., Clausen B.E., Yogev N. (2017). Dendritic cells as gatekeepers of tolerance. Semin. Immunopathol..

[8] Kapsenberg M.L. (2003). Dendritic-cell control of pathogen-driven T-cell polarization. Nat. Rev. Immunol..

[9] De Jong E.C., Smits H.H., Kapsenberg M.L. (2005). Dendritic cell-mediated T cell polarization. Springer Semin. Immunopathol..

[10] Randolph G.J., Angeli V., Swartz M.A. (2005). Dendritic-cell trafficking to lymph nodes through lymphatic vessels. Nat. Rev. Immunol..

[11] Friedl P., Gunzer M. (2001). Interaction of T cells with APCs: The serial encounter model. Trends Immunol..

[12] Alvarez D., Vollmann E.H., von Andrian U.H. (2008). Mechanisms and Consequences of Dendritic Cell Migration. Immunity.

[13] Kawai T., Akira S. (2011). Toll-like Receptors and Their Crosstalk with Other Innate Receptors in Infection and Immunity. Immunity.

[14] Geijtenbeek T.B.H., Gringhuis S.I. (2009). Signalling through C-type lectin receptors: Shaping immune responses. Nat. Rev. Immunol..

[15] Takeuchi O., Akira S. (2010). Pattern Recognition Receptors and Inflammation. Cell.

[16] Kawai T., Akira S. (2006). TLR signaling. Cell Death Differ..

[17] Wang Y., Huang G., Vogel P., Neale G., Reizis B., Chi H. (2012). Transforming growth factor beta-activated kinase 1 (TAK1)-dependent checkpoint in the survival of dendritic cells promotes immune homeostasis and function. Proc. Natl. Acad. Sci. USA.

[18] Powell J.D., Pollizzi K.N., Heikamp E.B., Horton M.R. (2012). Regulation of Immune Responses by mTOR. Annu. Rev. Immunol..

[19] Everts B., Amiel E., Huang S.C.C., Smith A.M., Chang C.H., Lam W.Y., Redmann V., Freitas T.C., Blagih J., Van Der Windt G.J.W. (2014). TLR-driven early glycolytic reprogramming via the kinases TBK1-IKKε supports the anabolic demands of dendritic cell activation. Nat. Immunol..

[20] Krawczyk C.M., Holowka T., Sun J., Blagih J., Amiel E., DeBerardinis R.J., Cross J.R., Jung E., Thompson C.B., Jones R.G. (2010). Toll-like receptor-induced changes in glycolytic metabolism regulate dendritic cell activation. Blood.

[21] Pearce E.J., Everts B. (2015). Dendritic cell metabolism. Nat. Rev. Immunol..

[22] Thwe P.M., Pelgrom L., Cooper R., Beauchamp S., Reisz J.A., D’Alessandro A., Everts B., Amiel E. (2017). Cell-Intrinsic Glycogen Metabolism Supports Early Glycolytic Reprogramming Required for Dendritic Cell Immune Responses. Cell Metab..

[23] Nouwen L.V., Everts B. (2020). Pathogens MenTORing Macrophages and Dendritic Cells: Manipulation of mTOR and Cellular Metabolism to Promote Immune Escape. Cells.

[24] Patente T.A., Pinho M.P., Oliveira A.A., Evangelista G.C.M., Bergami-Santos P.C., Barbuto J.A.M. (2019). Human dendritic cells: Their heterogeneity and clinical application potential in cancer immunotherapy. Front. Immunol..

[25] Escors D., Lopes L., Lin R., Hiscott J., Akira S., Davis R.J., Collins M.K. (2008). Targeting dendritic cell signaling to regulate the response to immunization. Blood.

[26] Hart G.W., Slawson C., Ramirez-Correa G., Lagerlof O. (2011). Cross talk between O-GlcNAcylation and phosphorylation: Roles in signaling, transcription, and chronic disease. Annu. Rev. Biochem..

[27] Shi J., Tomašič T., Sharif S., Brouwer A.J., Anderluh M., Ruijtenbeek R., Pieters R.J. (2017). Peptide microarray analysis of the cross-talk between O-GlcNAcylation and tyrosine phosphorylation. FEBS Lett..

[28] Dias W.B., Cheung W.D., Hart G.W. (2012). O-GlcNAcylation of kinases. Biochem. Biophys. Res. Commun..

[29] Butkinaree C., Park K., Hart G.W. (2010). O-linked β-*N*-acetylglucosamine (O-GlcNAc): Extensive crosstalk with phosphorylation to regulate signaling and transcription in response to nutrients and stress. Biochim. Biophys. Acta.

[30] Very N., Vercoutter-Edouart A.-S., Lefebvre T., Hardivillé S., El Yazidi-Belkoura I. (2018). Cross-Dysregulation of O-GlcNAcylation and PI3K/AKT/mTOR Axis in Human Chronic Diseases. Front. Endocrinol..

[31] Very N., Steenackers A., Dubuquoy C., Vermuse J., Dubuquoy L., Lefebvre T., El Yazidi-Belkoura I. (2018). Cross regulation between mTOR signaling and O-GlcNAcylation. J. Bioenerg. Biomembr..

[32] Biwi J., Biot C., Guerardel Y., Vercoutter-Edouart A.-S., Lefebvre T. (2018). The Many Ways by Which O-GlcNAcylation May Orchestrate the Diversity of Complex Glycosylations. Molecules.

[33] Lazarus M.B., Nam Y., Jiang J., Sliz P., Walker S. (2011). Structure of human O-GlcNAc transferase and its complex with a peptide substrate. Nature.

[34] Ma Z., Vosseller K. (2013). O-GlcNAc in cancer biology. Amino Acids.

[35] Ferrer C.M., Sodi V.L., Reginato M.J. (2016). O-GlcNAcylation in Cancer Biology: Linking Metabolism and Signaling. J. Mol. Biol..

[36] Lefebvre T., Guinez C., Dehennaut V., Beseme-Dekeyser O., Morelle W., Michalski J.C. (2005). Does O-GlcNAc play a role in neurodegenerative diseases?. Expert Rev. Proteomics.

[37] Hewagama A., Gorelik G., Patel D., Liyanarachchi P., Joseph McCune W., Somers E., Gonzalez-Rivera T., Strickland F., Richardson B., The Michigan Lupus Cohort (2013). Overexpression of X-Linked genes in T cells from women with lupus. J. Autoimmun..

[38] Hart G.W. (1997). Dynamic O-linked glycosylation of nuclear and cytoskeletal proteins. Annu. Rev. Biochem..

[39] Bond M.R., Hanover J.A. (2015). A little sugar goes a long way: The cell biology of O-GlcNAc. J. Cell Biol..

[40] Ortiz-Meoz R.F., Jiang J., Lazarus M.B., Orman M., Janetzko J., Fan C., Duveau D.Y., Tan Z.W., Thomas C.J., Walker S. (2015). A Small Molecule That Inhibits OGT Activity in Cells. ACS Chem. Biol..

[41] Chou T.Y., Hart G.W., Dang C. (1995). V c-Myc is glycosylated at threonine 58, a known phosphorylation site and a mutational hot spot in lymphomas. J. Biol. Chem..

[42] Bond M.R., Hanover J.A. (2013). O-GlcNAc Cycling: A Link Between Metabolism and Chronic Disease. Annu. Rev. Nutr..

[43] Hanover J.A., Chen W., Bond M.R. (2018). O-GlcNAc in cancer: An Oncometabolism-fueled vicious cycle. J. Bioenerg. Biomembr..

[44] Caldwell S.A., Jackson S.R., Shahriari K.S., Lynch T.P., Sethi G., Walker S., Vosseller K., Reginato M.J. (2010). Nutrient sensor O-GlcNAc transferase regulates breast cancer tumorigenesis through targeting of the oncogenic transcription factor FoxM1. Oncogene.

[45] De Jesus T., Shukla S., Ramakrishnan P. (2018). Too sweet to resist: Control of immune cell function by O-GlcNAcylation. Cell. Immunol..

[46] Machacek M., Slawson C., Fields P.E. (2018). O-GlcNAc: A novel regulator of immunometabolism. J. Bioenerg. Biomembr..

[47] He Y., Ma X., Li D., Hao J. (2017). Thiamet G mediates neuroprotection in experimental stroke by modulating microglia/macrophage polarization and inhibiting NF-κB p65 signaling. J. Cereb. Blood Flow Metab..

[48] Abramowitz L.K., Hanover J.A. (2018). T cell development and the physiological role of O-GlcNAc. FEBS Lett..

[49] Machacek M., Saunders H., Zhang Z., Tan E.P., Li J., Li T., Villar M.T., Artigues A., Lydic T., Cork G. (2019). Elevated O-GlcNAcylation enhances pro-inflammatory Th17 function by altering the intracellular lipid microenvironment. J. Biol. Chem..

[50] Wu J.-L., Chiang M.-F., Hsu P.-H., Tsai D.-Y., Hung K.-H., Wang Y.-H., Angata T., Lin K.-I. (2017). O-GlcNAcylation is required for B cell homeostasis and antibody responses. Nat. Commun..

[51] Chang Y.-H., Weng C.-L., Lin K.-I. (2020). O-GlcNAcylation and its role in the immune system. J. Biomed. Sci..

[52] Lee D.H., Kwon N.E., Lee W.-J., Lee M.-S., Kim D.-J., Kim J.H., Park S.-K. (2020). Increased O-GlcNAcylation of c-Myc Promotes Pre-B Cell Proliferation. Cells.

[53] Martin S.E.S., Tan Z.-W., Itkonen H.M., Duveau D.Y., Paulo J.A., Janetzko J., Boutz P.L., Törk L., Moss F.A., Thomas C.J. (2018). Structure-Based Evolution of Low Nanomolar O-GlcNAc Transferase Inhibitors. J. Am. Chem. Soc..

[54] Švajger U., Anderluh M., Jeras M., Obermajer N. (2010). C-type lectin DC-SIGN: An adhesion, signalling and antigen-uptake molecule that guides dendritic cells in immunity. Cell. Signal..

[55] Pustylnikov S., Sagar D., Jain P., Khan Z.K. (2014). Targeting the C-type Lectins-Mediated Host-Pathogen Interactions with Dextran. J. Pharm. Pharm. Sci..

[56] Leney A.C., Atmioui D.E., Wu W., Ovaa H., Heck A.J.R. (2017). Elucidating crosstalk mechanisms between phosphorylation and O-GlcNAcylation. Proc. Natl. Acad. Sci. USA.

[57] Van der Laarse S.A.M., Leney A.C., Heck A.J.R. (2018). Crosstalk between phosphorylation and O-GlcNAcylation: Friend or foe. FEBS J..

[58] Yang X., Qian K. (2017). Protein O-GlcNAcylation: Emerging mechanisms and functions. Nat. Rev. Mol. Cell Biol..

[59] Chatham J.C., Marchase R.B. (2010). Protein O-GlcNAcylation: A critical regulator of the cellular response to stress. Curr. Signal Transduct. Ther..

[60] Machacek M., Slawson C., Fields P.E. (2020). Friend or Foe? Opposing Functions of O-GlcNAc in Regulating Inflammation. J. Cell. Immunol..

[61] Zhang B., Zhou P., Li X., Shi Q., Li D., Ju X. (2017). Bitterness in sugar: O-GlcNAcylation aggravates pre-B acute lymphocytic leukemia through glycolysis via the PI3K/Akt/c-Myc pathway. Am. J. Cancer Res..

[62] Sukhbaatar N., Hengstschläger M., Weichhart T. (2016). mTOR-Mediated Regulation of Dendritic Cell Differentiation and Function. Trends Immunol..

[63] Sarbassov D.D., Guertin D.A., Ali S.M., Sabatini D.M. (2005). Phosphorylation and regulation of Akt/PKB by the rictor-mTOR complex. Science.

[64] Yang J., Cron P., Good V.M., Thompson V., Hemmings B.A., Barford D. (2002). Crystal structure of an activated Akt/Protein Kinase B ternary complex with GSK3-peptide and AMP-PNP. Nat. Struct. Biol..

[65] Koorella C., Nair J.R., Murray M.E., Carlson L.M., Watkins S.K., Lee K.P. (2014). Novel regulation of CD80/CD86-induced phosphatidylinositol 3-kinase signaling by NOTCH1 protein in interleukin-6 and indoleamine 2,3-dioxygenase production by dendritic cells. J. Biol. Chem..

[66] Van De Laar L., Van Den Bosch A., Boonstra A., Binda R.S., Buitenhuis M., Janssen H.L.A., Coffer P.J., Woltman A.M. (2012). PI3K-PKB hyperactivation augments human plasmacytoid dendritic cell development and function. Blood.

[67] Shi J., Wu S., Dai C., Li Y., Grundke-Iqbal I., Iqbal K., Liu F., Gong C.-X. (2012). Diverse Regulation of AKT and GSK-3β by O-GlcNAcylation in Various Types of Cells. FEBS Lett..

[68] Qiang A., Slawson C., Fields P.E. (2021). The Role of O-GlcNAcylation in Immune Cell Activation. Front. Endocrinol..

[69] Liu B., Salgado O.C., Singh S., Hippen K.L., Maynard J.C., Burlingame A.L., Ball L.E., Blazar B.R., Farrar M.A., Hogquist K.A. (2019). The lineage stability and suppressive program of regulatory T cells require protein O-GlcNAcylation. Nat. Commun..

[70] Nakahara T., Moroi Y., Uchi H., Furue M. (2006). Differential role of MAPK signaling in human dendritic cell maturation and Th1/Th2 engagement. J. Dermatol. Sci..

[71] Kneass Z.T., Marchase R.B. (2005). Protein O-GlcNAc modulates motility-associated signaling intermediates in neutrophils. J. Biol. Chem..

[72] Jiang M., Qiu Z., Zhang S., Fan X., Cai X., Xu B., Li X., Zhou J., Zhang X., Chu Y. (2016). Elevated O-GlcNAcylation promotes gastric cancer cells proliferation by modulating cell cycle related proteins and ERK 1/2 signaling. Oncotarget.

[73] Yang Y., Li X., Luan H.H., Zhang B., Zhang K., Nam J.H., Li Z., Fu M., Munk A., Zhang D. (2020). OGT suppresses S6K1-mediated macrophage inflammation and metabolic disturbance. Proc. Natl. Acad. Sci. USA.

[74] Van Elssen C.H.M.J., Vanderlocht J., Oth T., Senden-Gijsbers B.L.M.G., Germeraad W.T.V., Bos G.M.J. (2011). Inflammation restraining effects of prostaglandin E2 on natural killer-dendritic cell (NK-DC) interaction are imprinted during DC maturation. Blood.

[75] Švajger U., Rožman P.J. (2019). Synergistic Effects of Interferon-γ and Vitamin D3 Signaling in Induction of ILT-3highPDL-1high Tolerogenic Dendritic Cells. Front. Immunol..

[76] Anderson A.E., Swan D.J., Sayers B.L., Harry R.A., Patterson A.M., von Delwig A., Robinson J.H., Isaacs J.D., Hilkens C.M.U. (2009). LPS activation is required for migratory activity and antigen presentation by tolerogenic dendritic cells. J. Leukoc. Biol..

[77] Sun Q.-H., Wang Y.-S., Liu G., Zhou H.-L., Jian Y.-P., Liu M.-D., Zhang D., Ding Q., Zhao R.-X., Chen J.-F. (2020). Enhanced O-linked Glcnacylation in Crohn’s disease promotes intestinal inflammation. EBioMedicine.

[78] Li Y., Xie M., Men L., Du J. (2019). O-GlcNAcylation in immunity and inflammation: An intricate system (Review). Int. J. Mol. Med..

[79] Jonuleit H., Kühn U., Müller G., Steinbrink K., Paragnik L., Schmitt E., Knop J., Enk A.H. (1997). Pro-inflammatory cytokines and prostaglandins induce maturation of potent immunostimulatory dendritic cells under fetal calf serum-free conditions. Eur. J. Immunol..

