# Supplementary material for: Inhibition of O-GlcNAc Transferase Alters the Differentiation and Maturation Process of Human Monocyte Derived Dendritic Cells
Source: Cells. 2021 Nov 26;10(12):3312. doi: 10.3390/cells10123312 (PMC8699345; doi:10.3390/cells10123312)
Supplement: Supplementary file 1 [file cells-10-03312-s001.zip › cells-1377930-supplementary.pdf]

Inhibition of O-GlcNAc transferase alters the differentiation and maturation process of human monocyte derived dendritic cells

Matjaž Weiss [1], Marko Anderluh [1] and Martina Gobec [2,*]

[1] The Department of Pharmaceutical Chemistry, Faculty of Pharmacy, University of Ljubljana, 1000 Ljubljana, Slovenia; matjaz.weiss@ffa.uni-lj.si (M.W.), marko.anderluh@ffa.uni-lj.si (M.A.)
[2] The Department of Clinical Biochemistry, Faculty of Pharmacy, University of Ljubljana, 1000 Ljubljana, Slovenia
* Correspondence: martina.gobec@ffa.uni-lj.si; Tel.: +386-1-4769-636

CONTENTS

Methods..2

Viability assay...2

Results..2

Figure S1. Viability of moDCs...2

Figure S2. Characterisation of mTOR/AKT and MEK/ERK signalling pathways in the presence of OSMI-1. 3

Figure S3. Characterisation of specific DC surface markers in the presence of Vitamin D_3 together with IFN-γ (tolDCs). 4

Figure S4. Characterisation of specific DC surface markers in the presence of OSMI-1 during maturation. 4

Methods

Viability assay

Viability of moDCs was measured by PI staining (ThermoFischer Scientific). Briefly, 2 × 10^5 treated or untreated moDCs were stained by 2.0 µg/mL of PI for 5 min. Measurements were performed on Attune NxT flow cytometer (ThermoFisher Scientific).

Results

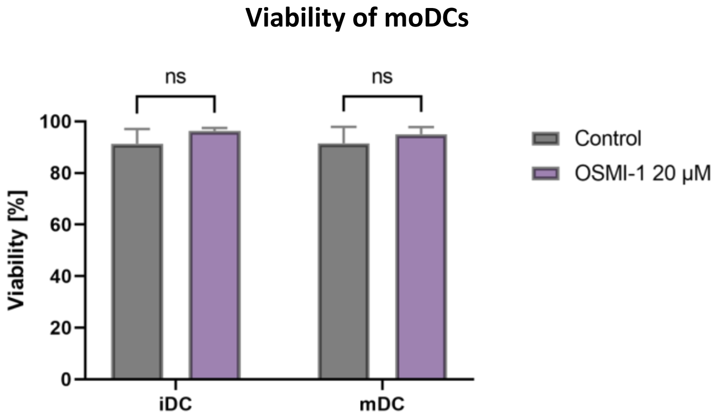

Figure S1. *Viability of moDCs.*

Viability of untreated or OSMI-1 treated immature and mature moDCs was assessed by PI. Results are presented as percentage of viable cells (PI negative) ± SD (4 different donors). Statistical significance between individual pairs was calculated using Student's t-test; p-value < 0.05 was considered statistically significant. iDCs, immature moDCs; mDCs, mature moDCs.

OGT inhibitor OSMI-1 affects the MEK/ERK and mTOR/AKT signalling axis in moDCs

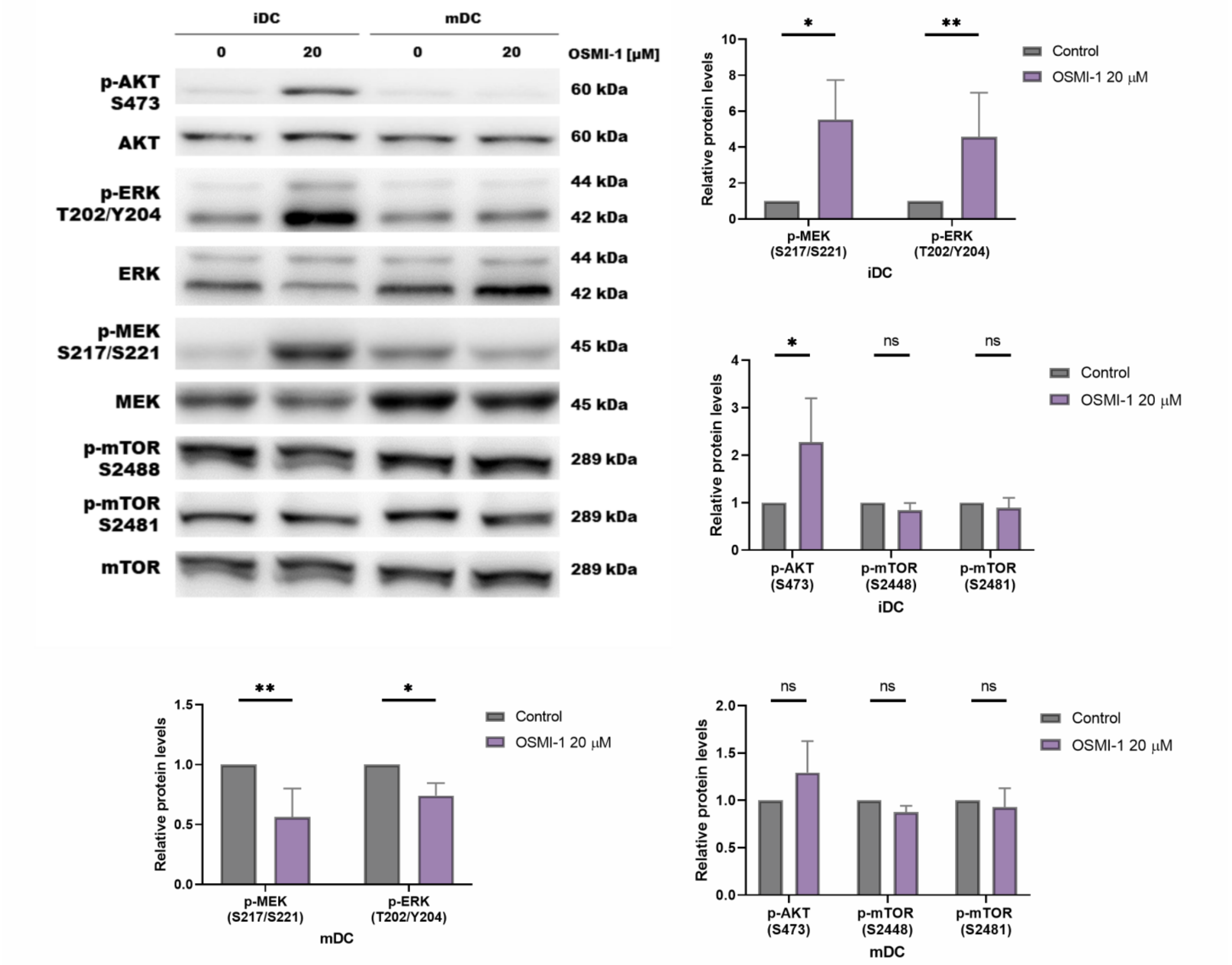

Figure S2. *Characterisation of mTOR/AKT and MEK/ERK signalling pathways in the presence of OSMI-1.* Representative immunoblots of phosphorylated ERK, MEK, AKT and mTOR, and their total expression in untreated or OSMI-1 treated immature (day 6) and mature moDCs (day 8). The results in charts are presented as a ratio of phosphorylated/total protein levels normalized to untreated moDCs ± SD (4-6 different donors). Statistical significance between individual pairs was calculated using Student's t-test; p-value < 0.05 was considered statistically significant. iDCs, immature moDCs; mDCs, mature moDCs.

Phenotype of tolDCs and mature OSMI-1-moDCs obtained from nontreated immature moDCs

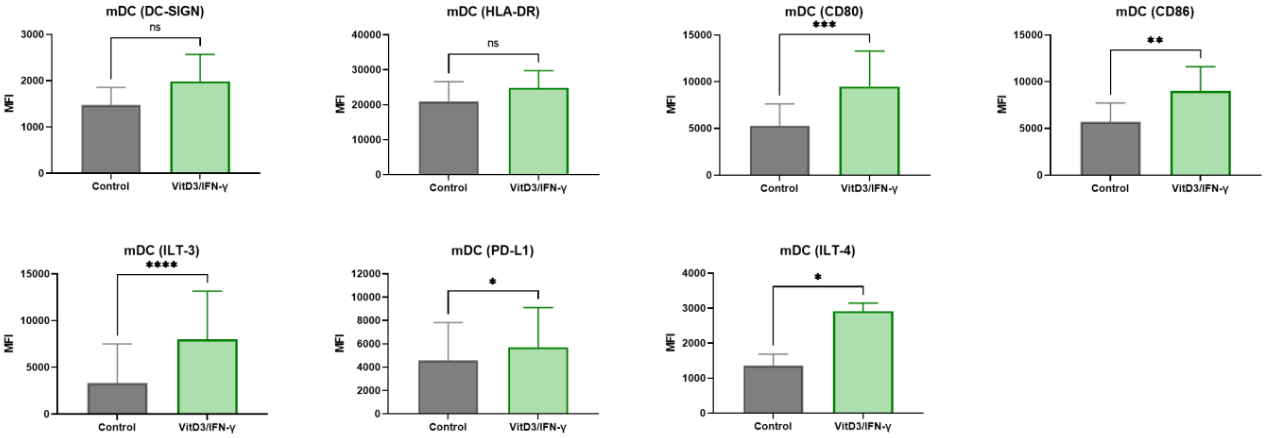

Figure S3. *Characterisation of specific DC surface markers in the presence of Vitamin D_3 together with IFN-γ (tolDCs).*

Shown are mean MFI values ± SD of n=6-8 independent experiments. tolDC were prepared from immature moDCs following 2 days incubation with the cytokine cocktail and the presence of Vitamin D_3 together with IFN-γ. Statistical significance between individual pairs was calculated using Student's t-test; p-value < 0.05 was considered statistically significant. iDCs, immature moDCs; mDCs, mature moDCs.

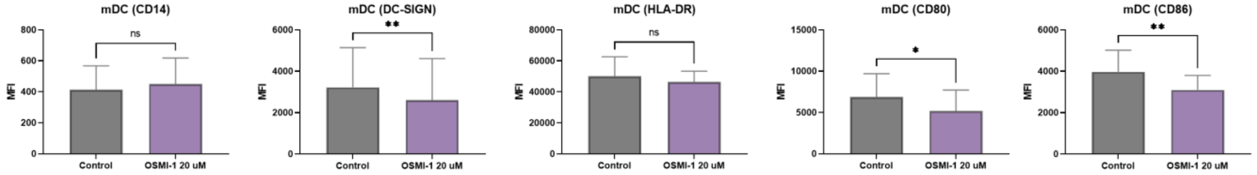

Figure S4. *Characterisation of specific DC surface markers in the presence of OSMI-1 during maturation.*

Shown are mean MFI values ± SD of n=6-8 independent experiments. Mature moDCs were prepared from control immature moDCs following 2 days incubation with the cytokine cocktail. The immature moDCs (control) were either untreated or treated with 20 µM of OSMI-1 for additional 2 days. Statistical significance between individual pairs was calculated using Student's t-test; p-value < 0.05 was considered statistically significant. iDCs, immature moDCs; mDCs, mature moDCs.
